# Development and application of *in-vivo* dose and time-resolved measurements for clinical application of ultra-high dose rate radiotherapy

**DOI:** 10.1016/j.phro.2026.100959

**Published:** 2026-03-30

**Authors:** Riccardo Dal Bello, Serena Psoroulas, Dominik Flückiger, Jerome Krayenbühl, Arvid Kemper, Rafael Kranzer, Benjamin Côté, Jens von der Grün, Panagiotis Balermpas, Matthias Guckenberger, Stephanie Tanadini-Lang

**Affiliations:** a Department of Radiation Oncology, University Hospital Zurich and University of Zurich, Zurich, Switzerland; b PTW-Freiburg, Freiburg, Germany; c Medscint Inc., Québec, QC, Canada

**Keywords:** Ultra-high dose rate, Flash effect, *In-vivo*, Time-resolved, Dosimetry

## Abstract

**Background and purpose:**

Ultra-high-dose-rate (UHDR) radiotherapy (RT) has emerged as a promising technique to widen the therapeutic window. However, clinical implementation is critically dependent on standardized dosimetry systems. This study reports on the integration of passive and active detectors for *in-vivo* dose monitoring in a clinical setting.

**Materials and methods:**

Four detector systems were evaluated for patient treatments with UHDR at a modified C-arm linear accelerator (linac) (9 MeV, dose per pulse 1.08 Gy, average dose rate 216 Gy/s): two passive systems − optically stimulated luminescence detectors (OSLDs) and radiochromic films − and two active systems − a scintillator and a diamond detector. The detectors were positioned on a dedicated 3D-printed mount or the patient’s skin to measure integral dose and time-resolved parameters. OSLDs were calibrated against a reference ion chamber and validated under varying conditions. Scintillator and diamond detectors recorded pulse-to-pulse and intra-pulse stability, respectively.

**Results:**

OSLDs provided reproducible dose reporting within ±5%, with precision improved through averaging multiple chips. The pulse-to-pulse stability measured with the scintillator was in agreement with the diamond detector measurements within ±1%. The diamond detector resolved intra-pulse stability and identified instabilities in case of poor beam tuning.

**Conclusions:**

The combined use of OSLDs, scintillator, diamond, and film detectors enabled robust and time-resolved recording of UHDR RT in a clinical trial setting. While resource-intensive, this approach ensured redundancy and precision, providing a feasible strategy for early clinical adoption.

## Highlights

• Seven patients were treated with an ultra-high dose rate electron beam.
• A dedicated *in-vivo* dosimetry approach was developed.
• The *in-vivo* measurements reported integral and time-resolved dose.

## Graphical abstract

**Figure:**
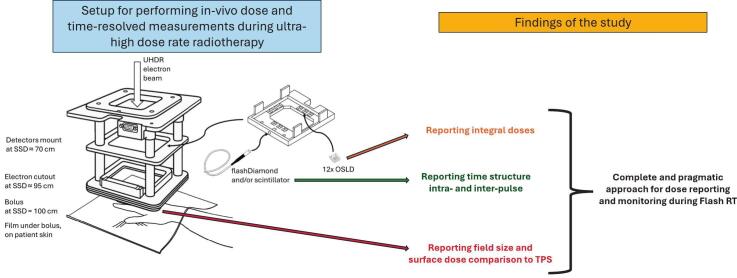

## 1 Introduction

The efficacy of radiotherapy (RT) is ultimately restricted by the tolerance of healthy tissues near the target. Ultra-high-dose-rate (UHDR) RT, i.e., average dose rates exceeding 40 Gy/s, was shown in preclinical studies to selectively spare normal tissue while preserving anti-tumour efficacy, a phenomenon known as the Flash effect [1], [2], [3]. A first patient received treatment with electron Flash RT in 2019 [4], followed by trials with both proton [5], [6] and electron beams [7], [8]. While clinical usage of UHDR RT is proceeding to collect clinical evidence on its feasibility, safety, efficacy and magnitude of the Flash effect in humans [9], [10]; the underlying biological mechanism is yet to be fully elucidated [11], [12], [13]. Several physical parameters define UHDR beam properties and limiting the description to the average dose rate may not be sufficient to determine whether the Flash effect will be triggered or not [14], [15]. According to experts’ consensus the minimal set of parameters to be reported extends beyond the requirements for conventional RT [16], such that future retrospective studies may have access to all the necessary information to model the Flash effect. Reported parameters should include not only the integral dose and the total delivery time, but also the beam pulse structure, detailing the pulse frequency, length and potentially intra-pulse stability. A common requirement to access such parameters is the use of active detectors with time-resolved capabilities that are compatible with UHDR.

The clinical delivery of UHDR RT has been made possible thanks to recent technological advances, which enabled UHDR stable delivery [17], [18], [19] and accurate detection [20], [21], [22], [23]. The latter, however, is still a developing research field. Several detector technologies have been proposed to cope with high dose rates and prototypes were successfully investigated. However, to date, none of the active UHDR-compatible detectors is marketed as a medical device. This applies to both linac-mounted internal detectors (e.g. ion chamber or current-converter) and to external detectors used for quality assurance and calibrations (e.g. scintillators or diamond). While several detector technologies have been demonstrated to be suitable for UHDR detection, their application in a clinical context is still scarce.

This study aimed to develop a practical and pragmatic methodology to employ UHDR-compatible detectors during treatments, which was investigated in a clinical setting. The investigation focused on the applicability of such methodology for integral and time-resolved dose reporting. By reporting our findings, we aimed to facilitate the employment of UHDR RT in a clinical context.

## 2 Materials and methods

### 2.1 Linear accelerator for ultra-high dose rate delivery

This study utilized the linac FLEX extension for the TrueBeam SN 1001 (Varian Medical Systems, a Siemens Healthineers company, Palo Alto, USA), a modified linear accelerator previously in clinical use until 2022 and then employed for the Flash-Skin I clinical trial (NCT06549439) [24]. Details of the conversion have been reported elsewhere [19], [25].

In brief, the UHDR extension increased dose per pulse (DPP) by using a thinner scattering foil, adjusting bending magnet strength to match conventional and UHDR beam qualities, optimizing electron gun output, and tuning radiofrequency power. A firmware patch disabled all servos except Automatic Frequency Control (AFC), with dose controlled via pulse counting at the Beam Generation and Monitoring (BGM) level. For safety, beam-on sessions in treatment mode were limited to 20 pulses. A delay between beam-on button press and beam delivery of 7.5 s was introduced for AFC stability. The linac was operated at 9 MeV throughout this study and the DPP for the reference field 10x10 cm^2^ square with SSD = 100 cm at Dmax in water was 1.08 Gy, leading to an average dose rate of 216 Gy/s [26]. Additional beam parameters are provided in the Supplementary Table S1.

### 2.2 Detectors and installation during clinical operation

**Fig. 1:**
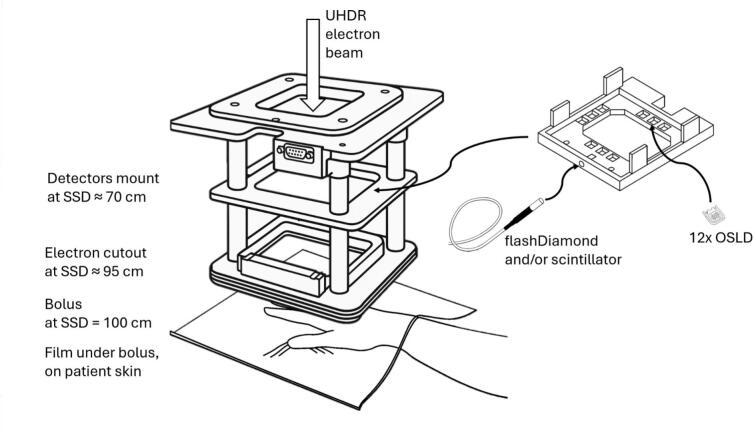
Schematic view of the setup used in the current study with positioning of detectors. Not to scale.

A dedicated approach was developed to record the dose for the Skin-Flash I trial. A set of detectors was employed and each detector had different objectives, including both integral and time-resolved dose recording. Their location with respect to the beamline and the patient was optimized to streamline workflow during clinical RT delivery, avoiding additional workload to the radiotherapy technologists (RTT). Multiple detectors were fixed on a dedicated 3D-printed mount on the electron tube outside the primary beam and at SSD ≈ 70 cm and, if a bolus was used, a film was located on the patient’s skin (Fig. 1). The purposes of the detectors were multiple and are summarized in Table 1.

**Table 1:** Overview of the detectors used in the current study.

Detector technology	Detector model	Readout	Purpose	Used during patient treatments	References
Diamond detector	PTW flashDiamond TM60025	Femto DHPCA-100 and Tektronix MSO54B operated at 6.25 GHz	Time-resolved intra-pulse measurements	Yes, but not available for all fractions	[20], [30], [31]
Organic scintillator	Medscint PRB-41	Medscint RP-100 operated at 1000 Hz	Time resolved pulse-to-pulse measurements	Yes, for all fractions	[22], [27]
Optically stimulated luminescence dosimeters	RadPro myOSLDchip dosimeter	RadPro myOSLDchip reader	Measurement of off-axis radiation and correlation with dose at isocenter	Yes, for all fractions	[28]
Self-developing dosimetry film	Ashland Gafchromic EBT3	Readout after 24 h with transmission measurements at Epson 12000XL flatbed scanner. 48-bit RGB at 150 dpi, processed with FilmQA Pro 4.0 (Ashland, Bridgewater, USA) and the triple channel analysis.	Integral dose measurement on patient skin for comparison with TPS	Yes, but only in presence of bolus	[32]
Ion chamber	PTW Advanced Markus TW34045	PTW Unidos	Reference dosimetry at isocenter in water	No	[35], [36]

Four detectors were characterized for use during patient treatments. Medscint’s scintillator Flash series detector (PRB-0041) was used to detect scattered radiation, perform pulse counting, and evaluate pulse-to-pulse beam stability [22], [27]. The scintillator detector was read out by Medscint’s Hyperscint RP-FLASH operated with a single channel, sampling frequency of 1 kHz and total measuring time of 5 s. Using these parameters created a series of acquisition windows of 1 ms which allows for single-pulse measurements, given that has a pulse repetition frequency (PRF) of 200 Hz and a pulse length of 4.2 μs. The timestamp of the detected signal was based on the laptop time on which the readout software was operated.

Twelve RadPro myOSLchip (optically stimulated luminescence detectors, OSLD) provided measurements of linac output during irradiation, which were converted to dose to isocenter and used as the primary dose reporting method. The signal stability over time was investigated in previous studies [28], [29]. The BeO OSLDs were bleached for 2 h to standardize sensitivity. The background signal was measured on the reader and chips were mounted on the 3D‑printed holder for the irradiation. A minimum 15 min wait allowed shallow trap recombination and signal stabilization. The signal was then read using the myOSLchip reader, and the OSLDs were finally bleached for 2 h to prepare them for subsequent use. The relative sensitivity between individual chips was determined during the factory calibration and not modified in this study.

A PTW flashDiamond detector connected to a preamplifier and digital oscilloscope was used to assess intra-pulse stability [20], [30], [31]. The transimpedance amplifier used was a Femto DHPCA-100 operated in DC, with gain 10^5^ V/A, bandwidth 14 MHz, which led to a nominal 10%–90% signal rise time due to the preamplifier of 25 ns. The signal was then read out by a Tektronix MSO54B operated in single-channel mode, a sampling frequency of 6.25 GHz, a trace length of 10 μs, and up to 100 traces stored. The trigger level was set at 300 mV, i.e. approximately 50% of the maximum signal. Single oscilloscope traces contained ∼2600 samples within each 4.2 μs long pulse delivered by the UHDR linac. The timestamp of the rise edge for the first trace was assigned to the same timestamp of the first pulse read by the scintillator.

Finally, an Ashland Gafchromic EBT3 film was placed on the patient’s skin beneath the bolus to measure the integral surface dose and field size [32]. The film calibration was performed at a conventional dose rate (CDR) linac in reference conditions and employed at UHDR as demonstrated suitable by previous investigations [33]. The readout was performed according to the vendor recommendations, allowing 24 h from the irradiation using an Epson Expression 12000XL flatbed scanner.

The model of the 3D-printed support for the detectors at SSD ≈ 70 cm is provided as open-source for further use [34]. The mount could host up to twelve OSLDs, three per direction ±X and ±Y. We refer to the four OSLDs along the major axis ±X/±Y as *Central*, the remaining as *Edges 1* (four chips in direction ±Y) and as *Edges 2* (four chips in direction ±X). The location of the OSLDs with respect to beam axis is reported in Supplementary Fig. S2.

Additionally, one ion chamber (PTW Advanced Markus [35], [36]) was used in the commissioning phase to characterize the electron tube-mounted detectors. It was used to perform reference dosimetry measurements at SSD = 100 cm and Dmax in water to cross-calibrate the tube-mounted detectors. In particular, the OSLDs mounted at SSD ≈ 70 cm were irradiated simultaneously and their readout was correlated to the dose reported by the ion chamber. The process was repeated 10 times on different days for the 10 cm square field and 10 times for a 10 × 6 cm^2^ rectangular field. The average value over the 10 repetitions was taken as the calibration to perform dose reporting with the OSLDs.

### 2.3 Verification of detectors performances

The OSLDs were the primary method for dose reporting, therefore an extended characterization of their performance was conducted against isocentre measurements. Their response was verified against field size, gantry angle (0°, 90°, 270°), number of pulses (from 8 to 12) and DPP (pulse length from 2.5 μs to 4.2 μs). Periodic quality assurance (QA) also aimed to verify that the calibration against the Advanced Markus remained stable within ±5% over time.

The scintillator and the diamond detector provided time-resolved measurements. The performance of the two detectors in pulse-to-pulse stability recording was compared with both detectors installed off-beam at SSD ≈ 70 cm. The scintillator readout provided an integrated value of the pulse dose, while the diamond signal was integrated within the full-width at half-maximum (FWHM) to obtain the pulse dose. The intra-pulse stability measurements performed with the diamond detector could not be benchmarked against any other detector presented in Table 1. The benchmark was performed utilizing the W-target of the linac to stop the electron beam by reading the target signal with the same electronics as used for the diamond. These measurements were destructive, i.e. the electron beam stopped on the W-target and did not reach the isocenter, therefore consecutive measurements were performed with the diamond (W-target retracted) and the W-target assuming stable linac performances, compatible with output stability within ±5% previously reported [19].

## 3 Results

**Fig. 2:**
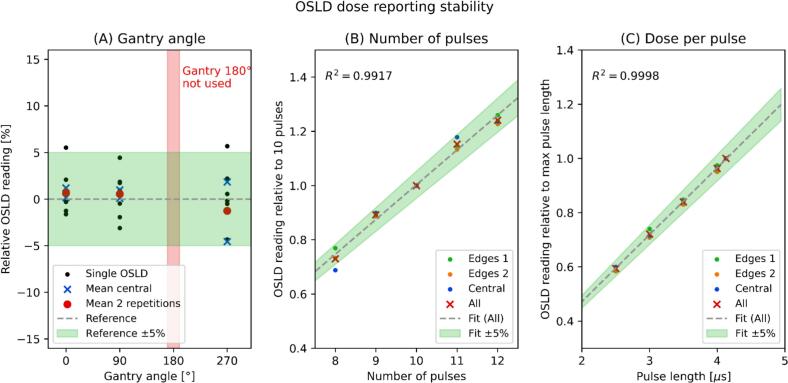
Stability of OSLDs for dose reporting under different conditions. The data reports measurements performed at different gantry angles (A, left), at varying number of pulses (B, center) and at varying dose-per-pulse controlled by varying the pulse length (C, right).

The OSLDs within the mount recorded approximately 1.5 Gy per each 1 Gy delivered at isocentre. The calibration for the electron tube 10x6 cm^2^ differed from the calibration of the 10x10 cm^2^ tube by 8.8% due to different location of the OSLDs with respect to the beam axis, while no significant differences (< 2%) were observed when varying the electron cutout for a given electron tube provided that the output factor correction for the field size was applied. The OSLDs readout was stable within ±5% with respect to the gantry angle (Fig. 2A). A single OSLD could present low precision with deviations up to 6.4%. Grouping the readout of the four central chips allowed improvement in precision by a factor $1/\sqrt{4}$. Further improvement with an additional factor $1/\sqrt{3}$ was achieved by averaging the dose reported by the Central, Edge 1 and Edge 2 OSLDs groups. This approach showed no deviation above ±5% when comparing the dose reported against the number of pulses (Fig. 2B) and the DPP (Fig. 2C). The QA program confirmed that the dose reported by the OSLDs remained stable within ± 5% over time against a reference detector [29].

**Fig. 3:**
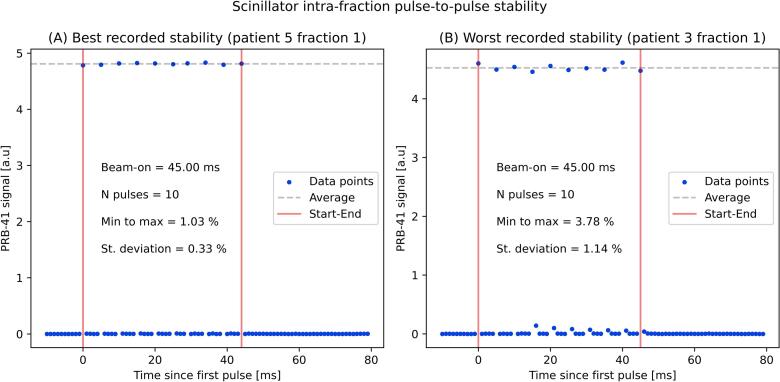
Measurements of the intra-fraction pulse-to-pulse stability assessed with the scintillator during patient treatments. The best (A, left) and worst (B, right) observed stabilities are reported.

**Fig. 4:**
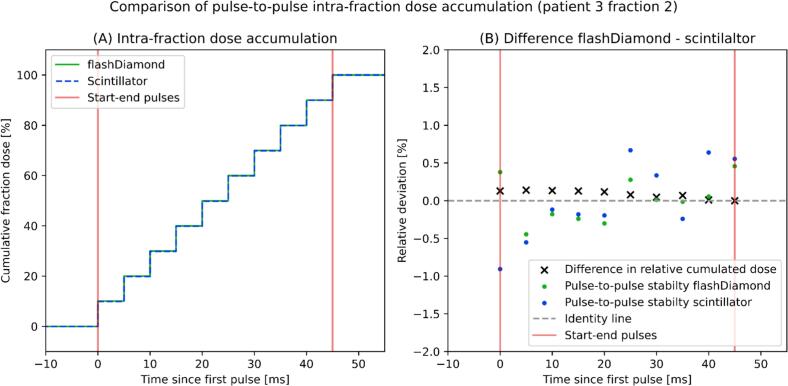
Pulse-to-pulse stability measured with flashDiamond compared to the stability measured with the scintillator. The cumulative dose (A, left) and the relative differences time-resolved at the pulse level (B, right) are shown.

The scintillator was shown to be suitable for measuring the pulse-to-pulse stability during patient treatments. At its location at SSD ≈ 70 cm off the primary beam it recorded approximately 5 cGy per each 1 Gy delivered at the isocenter, which also applied to the diamond. Fig. 3 shows two exemplary recordings performed during the trial, reporting 1.03% as the best (A) and 3.78% as the worst (B) recorded stability (min to max over average). The benchmark of these values with the diamond detector showed an overall agreement of the pulse-to-pulse stability measurements within ±1%. Fig. 4 shows an exemplary recording for a treated fraction in which both time-resolved detectors were available. The relative cumulative dose within a fraction reported by the detectors agreed within ±0.2%, while at the single pulse level no deviations outside ±1% were observed.

**Fig. 5:**
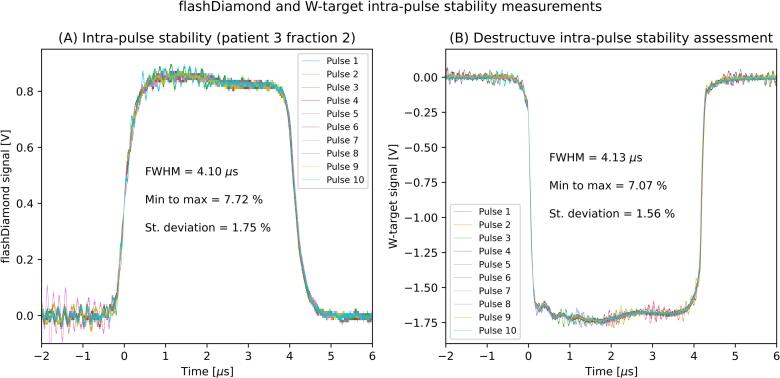
flashDiamond (A, left) and W-target (B, right) data for intra-pulse stability. While the flashDiamond data can be collected off-axis during a patient treatment, the W-target data requires inserting the W-target into the beam-line therefore performing a destructive measurement that cannot be performed during patient treatments.

The diamond detector could resolve intra-pulse structure. In consecutive measurements, the FWHM of investigated pulses showed agreement within ±1% when measured with either the diamond or the W-target. Fig. 5 shows exemplary data acquired during (A) and after (B) patient treatment, demonstrating an agreement of 1.3% of the intra-pulse stability measurement, calculated as ratio of minimum-to-maximum deviation over average within the interval of 0.4–3.7  μs (i.e., the central 80% of the pulse). The diamond detector further proved suitable for identifying intra-pulse instabilities caused by poor beam tuning, which were confirmed by measurements with the W-target (Supplementary Fig. S2).

## 4 Discussion

The present work demonstrated the feasibility of employing a set of four independent detectors during clinical ultra-high dose rate (UHDR) treatments in the context of the Skin-Flash I trial. The results demonstrated a robust and accurate method to report integral an time-resolved dose during patient treatments. The use of multiple devices was motivated by the fact that no standardized and clinically approved dosimetry system currently exists for UHDR radiotherapy [37]. In contrast to conventional treatments, where integrated linac chambers provide robust real-time output monitoring, UHDR beams still lack such solutions, making external dosimetry systems indispensable. Novel solutions such as beam-current transformers are being investigated and may provide linac-integrated detection of the beam structure [38], [39]. The approach adopted here combined four different detector technologies, each providing complementary information, enabling both integral dose verification and detailed time-resolved beam characterization, fulfilling existing recommendations [16].

Among the investigated devices, the OSLDs proved the most practical and were therefore used as the primary dose reporting method due to their ease of use in clinical conditions: they were compact, cable-free, simple to mount on the linac, and do not interfere with the RTT workflow. As passive detectors they remained in place during the fraction and did not disrupt patient positioning. OSLDs variability was minimized by averaging groups of twelve chips, achieving reproducibility within ±5% when benchmarked against reference detectors, which in turn were previously verified with external dosimetry audits [29]. It should be noted that with further investigation and validation, active devices such as scintillators or diamond may also serve as reliable detectors capable of recording both dose and temporal structure. However, such comparisons were beyond the scope of this study and the active detectors were only used for relative time-resolved measurements. In particular, the OSLDs were positioned closer to the central axis (Fig. 1) and had a greater signal (x30) than the active detectors in the setup utilized. It should be noted that the OSLDs required up to 30 min per fraction for readout. This went beyond what is typically required in CDR treatments, where the integrated ion chambers of the linac provide automated dose reporting. Although this strategy was feasible in the present clinical trial, it would be difficult to scale to routine patient UHDR treatments.

The scintillator and diamond detectors, while less convenient from an operational point of view, contributed with time-resolved data that OSLDs could not provide. Both required an external cable connection (coaxial for the diamond and optic fiber for the scintillator), which introduced challenges during patient setup. Positioning these cables demanded additional workload preparation by the medical physics team, however it was feasible and accomplished for all treated fractions within the trial for the scintillator. The diamond detector was available only for a sub-set of the fractions. Despite the challenges in their clinical usage, their utility lies in the possibility to detect in a time-resolved manner the UHDR beam and to obtain live feedback from the detector (OSLDs and film have delayed readout). The scintillator proved particularly useful for recording pulse-to-pulse stability, which was benchmarked against the diamond data. The scintillator detector used in this study was extensively characterized and found to be suitable for differentiating scintillating from Cherenkov light [22], [40], [41]. Since UHDR treatments cannot be characterized solely by average dose rate, such information is fundamental to ensuring reproducible delivery and accurate reporting of beam parameters. The diamond detector offered higher temporal resolution capable of resolving intra-pulse structures. Analysis of these structures revealed instabilities, such as those caused by poor beam tuning, which were subsequently confirmed by W-target measurements. Such measurements were critical to ensure correct beam-tuning within the clinical trial.

Radiochromic films played a different role. Their properties and robustness for UHDR dosimetry have been widely reported in the literature [42], [43], and our findings were in line with previous reports. The films were used to confirm the integral dose at each fraction, which satisfied gamma pass rates above 95% for 5%/3 mm against the treatment planning system. Given its already well-established role, we support its continued use because it remains a robust, low-interference method for surface dose verification in clinical settings.

The combination of OSLDs, scintillator, diamond and film represented a pragmatic and technically feasible solution to conduct UHDR dose recording during patient treatments. OSLDs ensured that patient dose reporting could be performed without disruption of the workflow. Scintillator and diamond detectors allowed the description of beam time structure at both the inter-pulse and intra-pulse level, providing unique insight into linac performance during radiotherapy delivery. Film provided robust confirmation of surface dose and treatment field size (Supplementary Fig. S3). While no single device was sufficient to cover all these aspects at once, their combined use ensured both redundancy and comprehensiveness.

This study also had limitations. All the detectors employed were not coupled to the UHDR linac through an active feedback system that could interrupt or extend the beam delivery. Future implementations may include beam-current transformers [38], [39]. This study focused on recording rather than actively monitoring and adapting the delivered dose. A further limitation of this study lies in the selection of OSLDs as the primary dose reporting system. While justified by their practicality, this choice led to a lack of investigation of the potential of scintillators or diamond detectors as dose reporting devices. Future work should investigate these possibilities, as merging integral dose verification with time-resolved monitoring into a single device could simplify workflows. Moreover, OSLDs may require a beam-quality correction if employed with different energies [44].

In conclusion, this study presented the development of a methodology to employ *in-vivo* time-resolved and dose-reporting detectors for UHDR radiotherapy. The study showed successful application during patient treatments. The dose reporting was achievable with a ±5% precision and the pulse-to-pulse stability measurements within ±1%. The data presented supports the use of this methodology for future UHDR clinical applications.

## CRediT authorship contribution statement

**Riccardo Dal Bello:** Conceptualization, Data curation, Formal analysis, Funding acquisition, Investigation, Methodology, Project administration, Software, Visualization, Writing – original draft. **Serena Psoroulas:** Conceptualization, Data curation, Investigation, Methodology, Writing – review & editing. **Dominik Flückiger:** Data curation, Formal analysis, Methodology, Writing – review & editing. **Jerome Krayenbühl:** Supervision, Validation, Writing – review & editing. **Arvid Kemper:** Data curation, Investigation, Methodology, Writing – review & editing. **Rafael Kranzer:** Resources, Supervision, Validation, Writing – review & editing. **Benjamin Côté:** Data curation, Investigation, Methodology, Writing – review & editing. **Jens von der Grün:** Investigation, Writing – review & editing. **Panagiotis Balermpas:** Investigation, Writing – review & editing. **Matthias Guckenberger:** Funding acquisition, Resources, Supervision, Validation, Writing – review & editing. **Stephanie Tanadini-Lang:** Conceptualization, Funding acquisition, Resources, Supervision, Validation, Writing – review & editing.

## Declaration of competing interest

The authors declare the following financial interests/personal relationships which may be considered as potential competing interests: The clinical trial was supported by a research grant by Siemens Healthineers.
