# Supplementary material for: Development and application of in-vivo dose and time-resolved measurements for clinical application of ultra-high dose rate radiotherapy
Source: Phys Imaging Radiat Oncol. 2026 Mar 30;38:100959. doi: 10.1016/j.phro.2026.100959 (PMC13085076; doi:10.1016/j.phro.2026.100959)
Supplement: Supplementary Data 1 [file mmc1.pdf]

Supplementary data

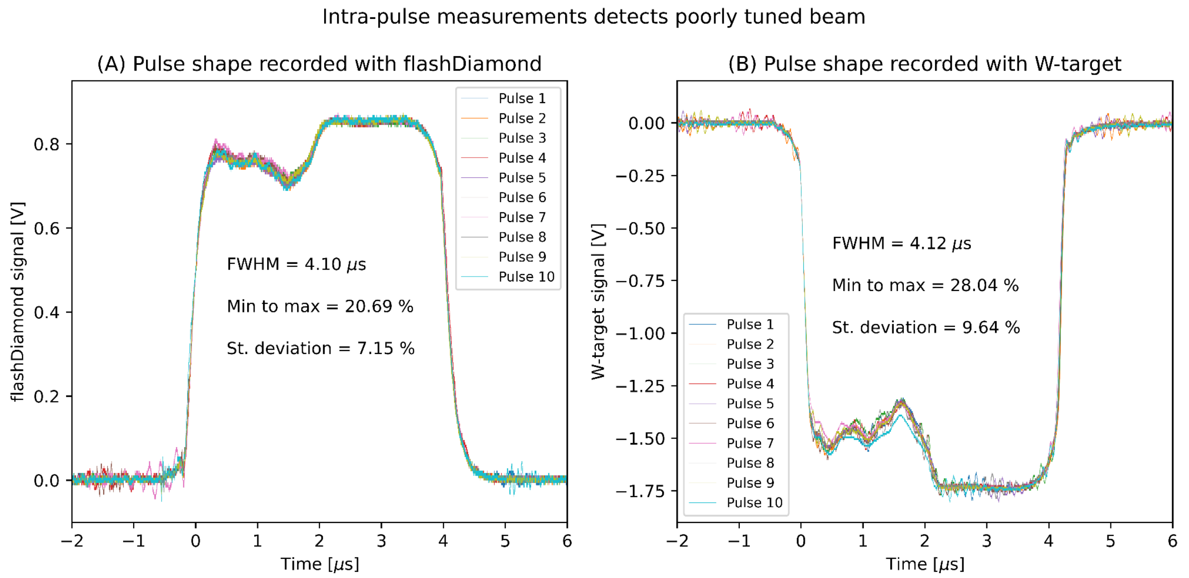

Supplementary Figure S1. FlashDiamond (A, left) and W-target (B, right) data collected during beam-tuning procedures in presence of a poorly tuned beam. The pulses present instability in the first half due to AFC sub-optimal tuning. Both methods (A) and (B) detect the intra-pulse dose variations.

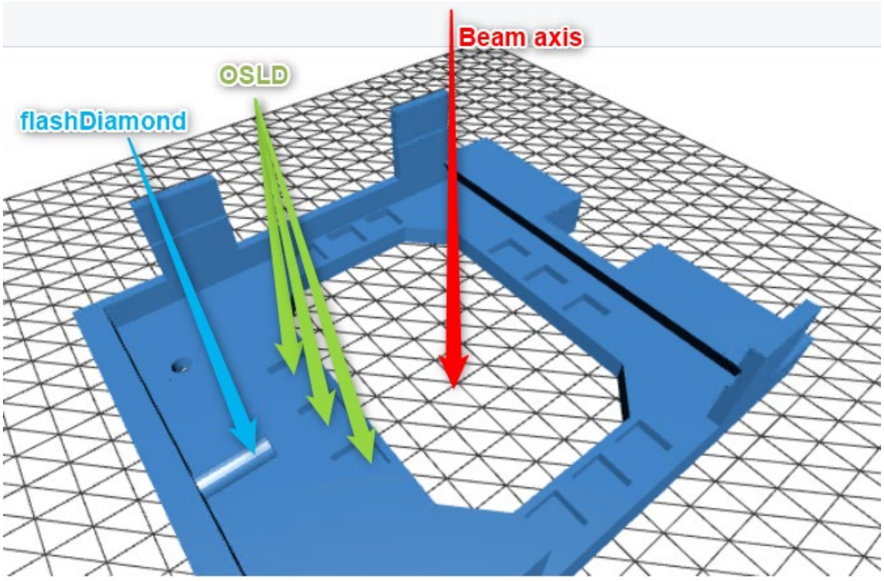

Supplementary Figure S2. Relative position of the detectors on the 3D printed support and the beam axis.

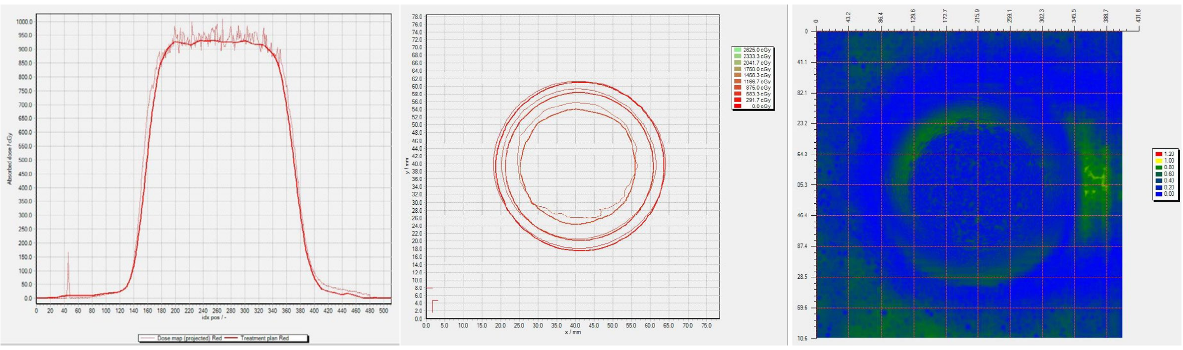

Supplementary Figure S3. Exemplary pre-treatment PSQA film measurements. Left: the comparison between TPS calculated dose (solid line) and film measurements (dashed line) is shown. The doses are shown along a left-right profile passing by the central axis for a 4 cm round (diameter) field. Centre: isodose lines 2D comparison. Right: gamma analysis 2%/2mm.

Quantity	Abbreviation	Values
**Minimal reporting**		
General description		
Device name	FLEX extension for TrueBeam v2.7.5	
Accelerator type	Electron linac	
Dose delivery technique	Scattering and collimation	
Traceability and dosimetry code of practice used	Detectors cross calibrated at conventional dose rate to a PTW Roos chamber, which was calibrated in the Swiss primary standard laboratory METAS. Reference dosimetry performed according to SSRMP recommendation nr. 10.	
Additional key information about delivery	None	
Preclinical: Biological system(s), model(s), endpoint(s)<br><br>Clinical: Site, diagnosis, stage, cohort characteristics	Human patients part of the Skin-Flash I trial. Condition: metastatic melanoma. Location: cutaneous or sub-cutaneous. Primary endpoint: skin toxicity grade 3 or higher.	
Additional key information (including imaging) about irradiated systems/models/patients	Imaging was performed prior to every treatment. Patient positioning was performed with the help of the TrueBeam CBCT. Immobilization and delivery used the same equipment used in conventional radiotherapy practice. Treatments were performed at isocenter (SSD = 100 cm) as in conventional radiotherapy practice and also the same electron tubes were used.	
Non-temporal beam parameters		
Radiation type and nominal beam energy	E	9 MeV electrons
Beam dose at reference point or volume	D_{beam}	9 Gy / fx
Reference point or volume specification[%]	P_{ref} or V_{ref}	The prescribed dose of 9 Gy was used to define the planning constraints for the PTV:<br><br>Coverage: D95% > 95% (tolerance D90% > 80%)<br><br>Maximum: D2% < 107% (tolerance: D2% < 115%)<br><br>Therefore, we report the dose as PTV prescribed dose
Source-to-surface distance	SSD	100 cm to the top of the bolus
Field size	FS	Multiple field sizes used. See main manuscript
Temporal beam structure parameters		

Pause before next beam	ΔT_{beam}	3 fractions delivered with a regimen of 2 fx/week
Beam-on time	T_{beam}	$(\#_{pulse\,-\,1})$ x 5 ms
Number of pulses for beam[§]	$\#_{pulse}$	Multiple pulses number used. See main manuscript.
Pulse length[§]	t_{pulse}	4.2 µs
Pulse repetition frequency[§]	*PRF*	200 Hz
Pulse charge[§]	Q_{pulse}	Approx 500 nC measured by inserting the photon bremsstrahlung target along the beam
Number of bunches per pulse [§]	$\#_{bunch}$	-
Bunch length[§]	t_{bunch}	-
Bunch repetition frequency[§]	*BRF*	-
Bunch charge[§]	Q_{bunch}	-
**Optimal reporting**		
Derived and additional parameters		
Average dose rate at reference point	ADR	216 Gy/s
Instantaneous dose rate at reference point	IDR	2.4 x 10^5 Gy/s
Dose per pulse	DPP	1.08 Gy/pulse
Representative 2D dose distribution of beam, PDD and lateral profiles	-	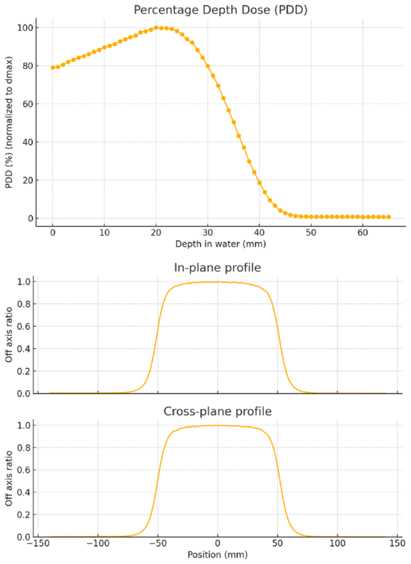
ADR-volume histograms of beam for relevant structures	-	-

Supplementary table S1. Beam data reporting as recommended by the ESTRO guideline Garibaldi C, Beddar S, Bizzocchi N, Böhlen TT, Iliaskou C, Moeckli R, et al. Minimum and optimal requirements for a safe clinical implementation of ultra-high dose rate radiotherapy: A focus on patient's safety and radiation protection. Radiother Oncol. 2024;196:110291.
